# Influence of Streptococcus pneumoniae Within-Strain Population Diversity on Virulence and Pathogenesis

**DOI:** 10.1128/spectrum.03103-22

**Published:** 2022-12-12

**Authors:** Laura C. Jacques, Angharad E. Green, Thomas E. Barton, Murielle Baltazar, Julia Aleksandrowicz, Rong Xu, Erwan Trochu, Aras Kadioglu, Daniel R. Neill

**Affiliations:** a Department of Clinical Infection, Microbiology and Immunology, University of Liverpool, Liverpool, United Kingdom; Emory University School of Medicine

**Keywords:** *Streptococcus pneumoniae*, evolution, infectious disease, population genetics, virulence

## Abstract

The short generation time of many bacterial pathogens allows the accumulation of *de novo* mutations during routine culture procedures used for the preparation and propagation of bacterial stocks. Taking the major human pathogen Streptococcus pneumoniae as an example, we sought to determine the influence of standard laboratory handling of microbes on within-strain genetic diversity and explore how these changes influence virulence characteristics and experimental outcomes. A single culture of S. pneumoniae D39 grown overnight resulted in the enrichment of previously rare genotypes present in bacterial freezer stocks and the introduction of new variation to the bacterial population through the acquisition of mutations. A comparison of D39 stocks from different laboratories demonstrated how changes in bacterial population structure taking place during individual culture events can cumulatively lead to fixed, divergent change that profoundly alters virulence characteristics. The passage of D39 through mouse models of infection, a process used to standardize virulence, resulted in the enrichment of high-fitness genotypes that were originally rare (<2% frequency) in D39 culture collection stocks and the loss of previously dominant genotypes. In the most striking example, the selection of a <2%-frequency genotype carrying a mutation in *sdhB*, a gene thought to be essential for the establishment of lung infection, was associated with enhanced systemic virulence. Three separately passaged D39 cultures originating from the same frozen stocks showed considerable genetic divergence despite comparable virulence.

**IMPORTANCE** Laboratory bacteriology involves the use of high-density cultures that we often assume to be clonal but that in reality are populations consisting of multiple genotypes at various abundances. We have demonstrated that the genetic structure of a single population of a widely used Streptococcus pneumoniae strain can be substantially altered by even short-term laboratory handling and culture and that, over time, this can lead to changes in virulence characteristics. Our findings suggest that caution should be applied when comparing data generated in different laboratories using the same strain but also when comparing data within laboratories over time. Given the dramatic reductions in the cost of next-generation sequencing technology in recent years, we advocate for the frequent sampling and sequencing of bacterial isolate collections.

## INTRODUCTION

The nutrient-rich environments of microbiological agar and broth support high rates of bacterial growth. Short generation times lead to the rapid accumulation of genetic diversity through *de novo* mutations that arise during error-prone chromosomal replication. Methodologies employed in the preparation and propagation of laboratory bacterial stocks are not associated with strong selective pressures but have the potential to impact the genetic structure of bacterial populations through sampling bias and population bottlenecks. The processes of recovering bacteria from frozen stocks, selecting single colonies or plate sweeps from growth on agar, and producing high-density liquid cultures allow genetic drift to influence genotype frequencies within bacterial populations. Over time, these practices may alter within-strain genetics, as suggested by the substantial laboratory-to-laboratory divergence in the genome sequence of the same reference strain of Pseudomonas aeruginosa (1).

In an experimental evolution study with the human pathogen Streptococcus pneumoniae, we identified more than 30 single nucleotide polymorphisms (SNPs) at a frequency of 2% or higher in our long-term freezer stocks of the widely used laboratory strain D39 (2). While no single SNP was found at a population frequency of >20% in these stocks, it was notable that many low-frequency variants later expanded or became fixed during experimental evolution. Rare variants can influence experimental outcomes if they are associated with a fitness benefit or if bottlenecking events enrich them as a proportion of the total population. It follows that changes occurring during culture, in either the preparation of bacteria for experimental assays or the onward propagation of frozen stocks, might similarly impact downstream studies.

Passaging pathogens through animal infection models as a means of standardizing or selecting for virulence has been practiced since at least the 1800s, including by Louis Pasteur during his work with rabies virus. Single or serial animal passage remains a widely used technique for handling viral pathogens (3–5) but has also been used for bacterial and fungal species (6–8). For S. pneumoniae, the practice involves the intraperitoneal (i.p.) administration of high-density cultures to mice, followed by the isolation of bacteria from the blood (9). Although this approach demonstrably selects for virulence, it is not clear why this is the case. The *in vivo* environment is associated with strong selective pressures from the immune system and challenges in nutrient acquisition, so passage may select high-fitness genotypes from the inoculum. The natural route for the development of pneumococcal sepsis, secondary to respiratory infection, is associated with tight bottlenecks (10, 11). However, the process of the intraperitoneal administration of high-density cultures used during passage bypasses many of these bottlenecks, potentially reducing the influence of genetic drift. Alternatively, or perhaps additionally, passage may standardize virulence by inducing environmentally triggered phenotypic changes in the bacterial population, such as those associated with the epigenetic modifications that occur during phase variation (12, 13). The ability of pneumococci to reversibly switch between opaque and transparent colony morphologies is under environment-specific control (14), with the opaque phenotype particularly being associated with systemic infection (15).

We sought to determine whether laboratory handling practices might shape the genetics of a single strain of Streptococcus pneumoniae and to define how these changes influence bacterial virulence. Culture on agar, or in nutrient broth, was associated with little change in the total SNP numbers relative to those that already existed in the frozen stocks of our culture collection. However, culture was associated with shifts in the SNP frequency and the loss and gain of polymorphisms. That such changes in population structure during individual culture events can cumulatively lead to fixed change and the divergence of strains was apparent from the comparison of six S. pneumoniae D39 stocks from our culture collection, which were acquired from different laboratories across the world over the last 3 decades. We identified between 4 and 24 fixed, nonsynonymous SNPs in the six D39 stocks relative to the D39 reference strain from the National Collection of Type Cultures. These genetic changes influenced bacterial phenotypes, including the growth rate, toxin production, and virulence in a mouse pneumonia model.

Next, we compared nonpassaged and mouse-passaged D39 prepared from the same long-term stock in our culture collection. Passage through an infection model selects for virulence by enriching for high-fitness genotypes and removing low-fitness genotypes. Three independently performed passages yielded stocks of comparable virulence, but each stock had a unique population structure with few shared, nonfixed mutations.

Our findings suggest that we should apply caution when comparing data generated by different laboratories using the same strain and when comparing data within a single laboratory over time. Given the dramatic reductions in the cost of next-generation sequencing technology in recent years, we advocate for the frequent sampling and population-level sequencing of bacterial isolate collections. Where animal passage for the purpose of standardization of virulence is necessary, a single passaged stock of a defined population structure should be used for the duration of an individual project.

## RESULTS

### Laboratory culture of Streptococcus pneumoniae enriches previously low-frequency genotypes.

**FIG 1:**
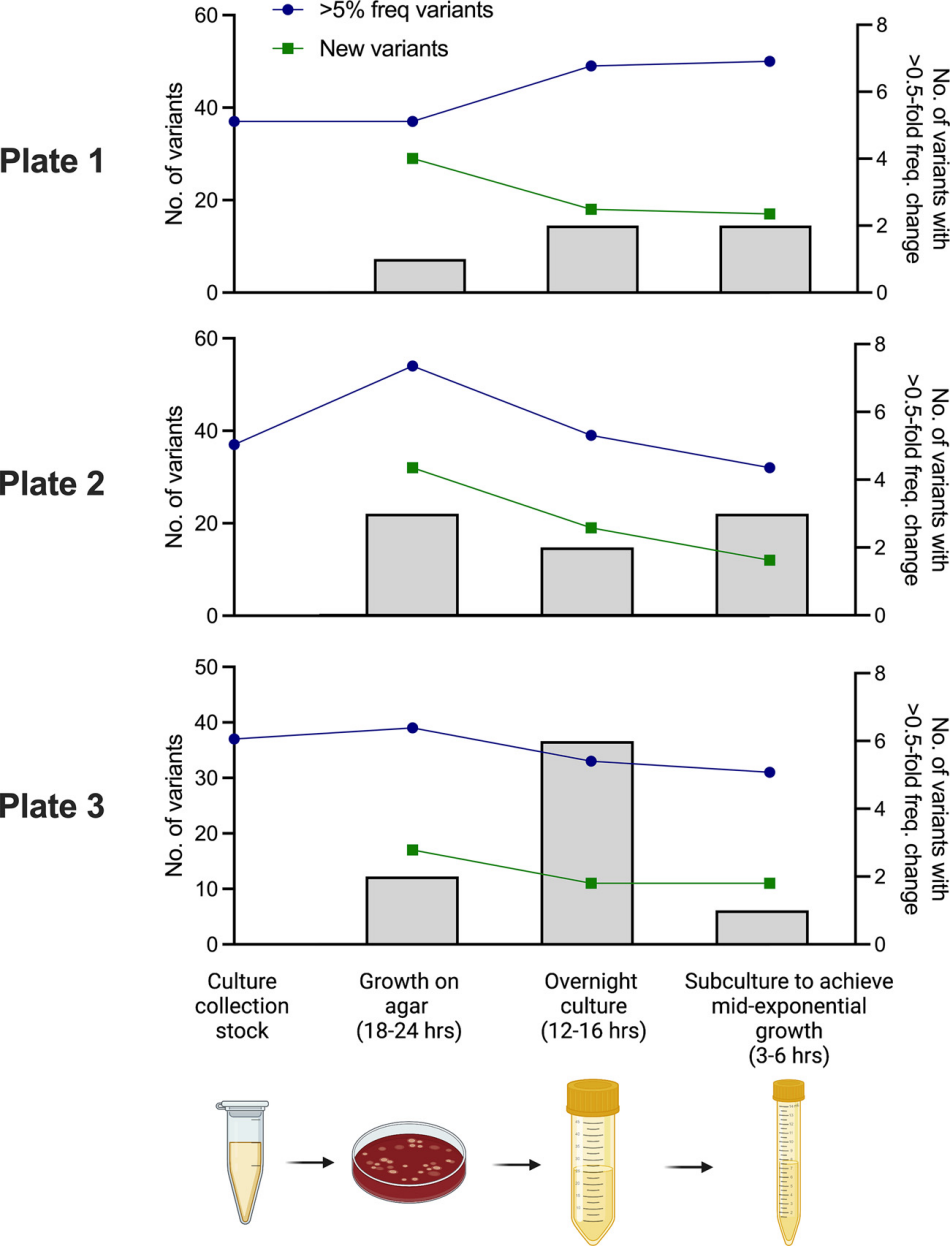
Changes in variant frequencies during culture. Variants are defined as synonymous or nonsynonymous single nucleotide polymorphisms, insertions, or deletions. Total variant numbers were determined from frozen culture collection stocks of D39N (leftmost points on the graphs) after culture overnight on agar (second points from the left), followed by liquid culture overnight (third points from the left) and, finally, subculture to mid-exponential phase (rightmost points). The experiment was conducted in parallel with three different cultures, all originating from a single frozen stock. Variant calling was performed using Breseq. Blue lines indicate the number of variants present at a frequency of >5% in the total population at each culture step. Green lines show the number of new variants arising, or first detected, at each culture step. Histograms show the number of variants with a >0.5-fold change in the frequency between individual culture steps (right axis).

Bacterial culture collections are maintained as frozen stocks in glycerol or coated onto the surface of ceramic beads. When required for experimental work, a frozen stock sample is typically streaked onto agar plates and incubated for at least 12 h. After this, bacteria are sampled as individual colony picks, sweeps of several colonies, or whole-plate scrapes and either used directly or following further expansion in broth. These are routine practices, undertaken in nutrient-rich culture environments presumed to be of low selective pressure. However, there are several bottlenecks during the process, offering the opportunity for genetic drift to influence bacterial population structure. From a single frozen bead stock tube of S. pneumoniae D39N, for which we have a recent consensus genome sequence and population sequencing data (2), we prepared three blood agar plates, each streaked with a single bead. After growth overnight, a sweep of colonies from each plate was used to prepare three separate broth cultures grown overnight. These cultures were then subcultured into fresh medium and incubated for a further 4 to 6 h to obtain high-density bacterial cultures at mid-exponential phase. These steps mimic those used for the preparation of bacteria for phenotypic assays, which variously call for bacteria sampled from agar (16), bacteria grown overnight (17), or mid-exponential-phase cultures (18). Short-read bacterial population sequence data derived from each step of the culture process were used to identify mutations that arose during growth or that changed in frequency during culture. The total number of SNPs in each population did not change dramatically during growth on agar or in broth (Fig. 1). Thirty-seven SNPs, at a population frequency of between 5 and 20%, were previously identified in our D39N bead stocks (2). In each of the three independently cultured populations here, the total number of SNPs did not exceed 54 or drop below 31 at any point during the growth processes. Of the 166 unique SNPs identified across all three experiments, 121, spread across 77 genes, were nonsynonymous (see Data Sets S1 to S3 in the supplemental material). However, few of these were shared with the bead stocks, suggesting the substantial loss and gain of low-frequency variants during culture (Fig. 1). Changes in the frequencies of individual mutations between culture steps were highly variable, with many remaining at consistent frequencies but others undergoing substantial expansion or contraction. In total, we observed 22 instances in which mutation frequencies increased or decreased by more than 0.5-fold during individual culture steps (Fig. 1). Over time, processes used for the onward propagation of stocks might therefore lead to the introduction of new variation and the expansion or contraction of existing genotypes in the population, contributing to phenotypic change.

### D39 stocks with a recent common origin display substantial genetic divergence.

**FIG 2:**
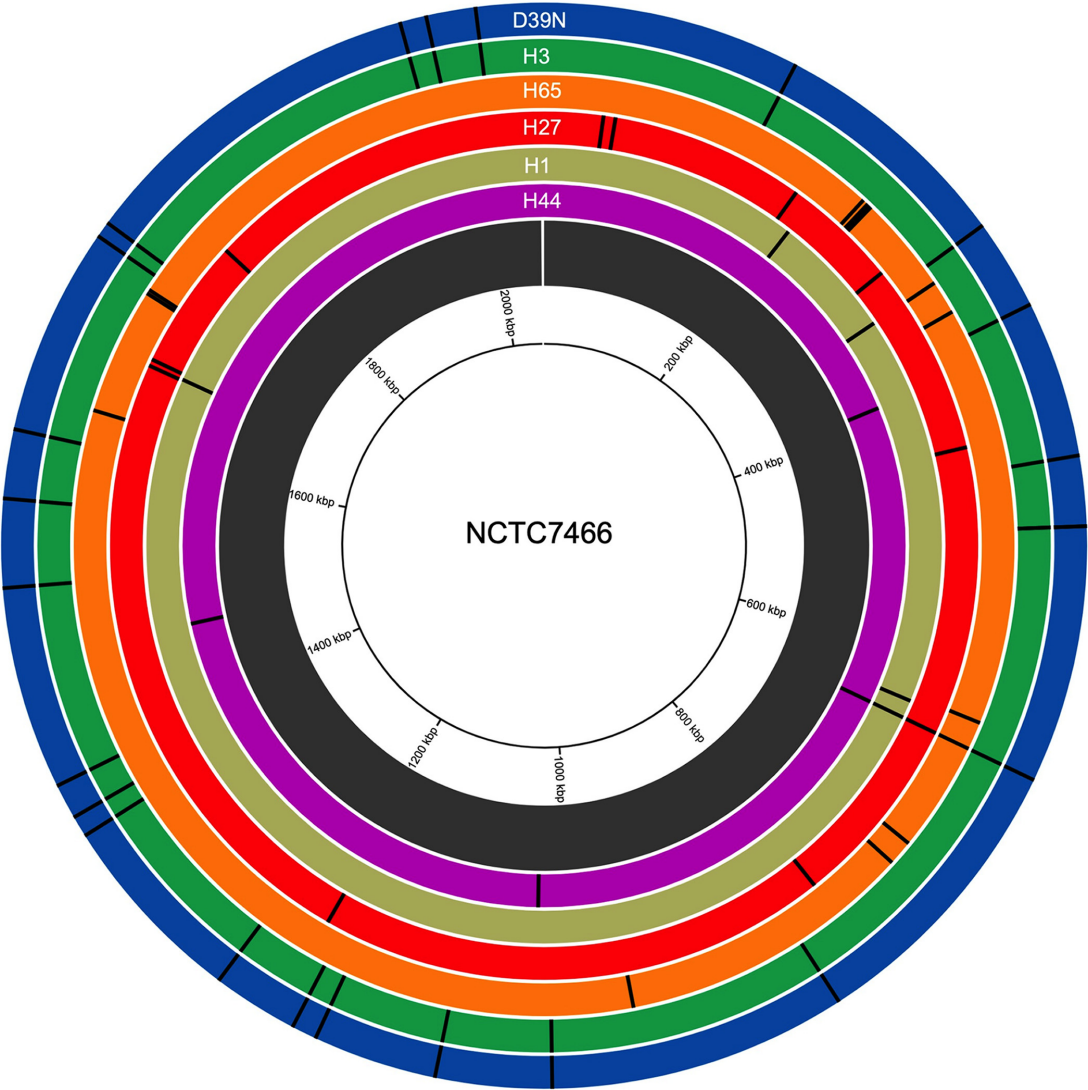
Nonsynonymous single nucleotide polymorphisms in S. pneumoniae D39 stocks acquired from different laboratories. Nonsynonymous SNPs are shown as black lines, indicating the genome position. Each track represents an individual D39 stock, and SNPs were identified using Breseq, using NCTC7466 D39 as the reference strain. Strains are organized by relatedness to NTCT7466, with the most distantly related strain (D39N) on the outermost track. Circa software (OMGenomics) was used for data visualization.

We had six D39 stocks in our culture collection, which were acquired from different laboratories over the past 3 decades. Mapping short-read sequence reads from these stocks against the genome of the National Collection of Type Cultures D39 strain (NCTC7466) revealed that all six D39 stocks had acquired fixed, nonsynonymous mutations (Fig. 2, Table 1, and Data Set S4). We identified between 4 and 24 fixed, nonsynonymous SNPs per D39 stock. All six D39 stocks shared a mutation in *spxB*, encoding the hydrogen peroxide-producing pyruvate oxidase, suggesting a common laboratory origin rather than independent strain acquisition from the NCTC. The effect of this mutation on peroxide production appeared minimal, as only one D39 strain showed evidence of significantly altered H_2_O_2_ production relative to NCTC7466 (Fig. S1), suggesting that the phenotype may not be a direct result of the *spxB* mutation. Genetically, D39N and H3 were most dissimilar to NCTC7466 but were identical to one another, while H44 was most similar to the type strain.

### Altered phenotypes associated with culture-acquired mutations in D39.

**FIG 3:**
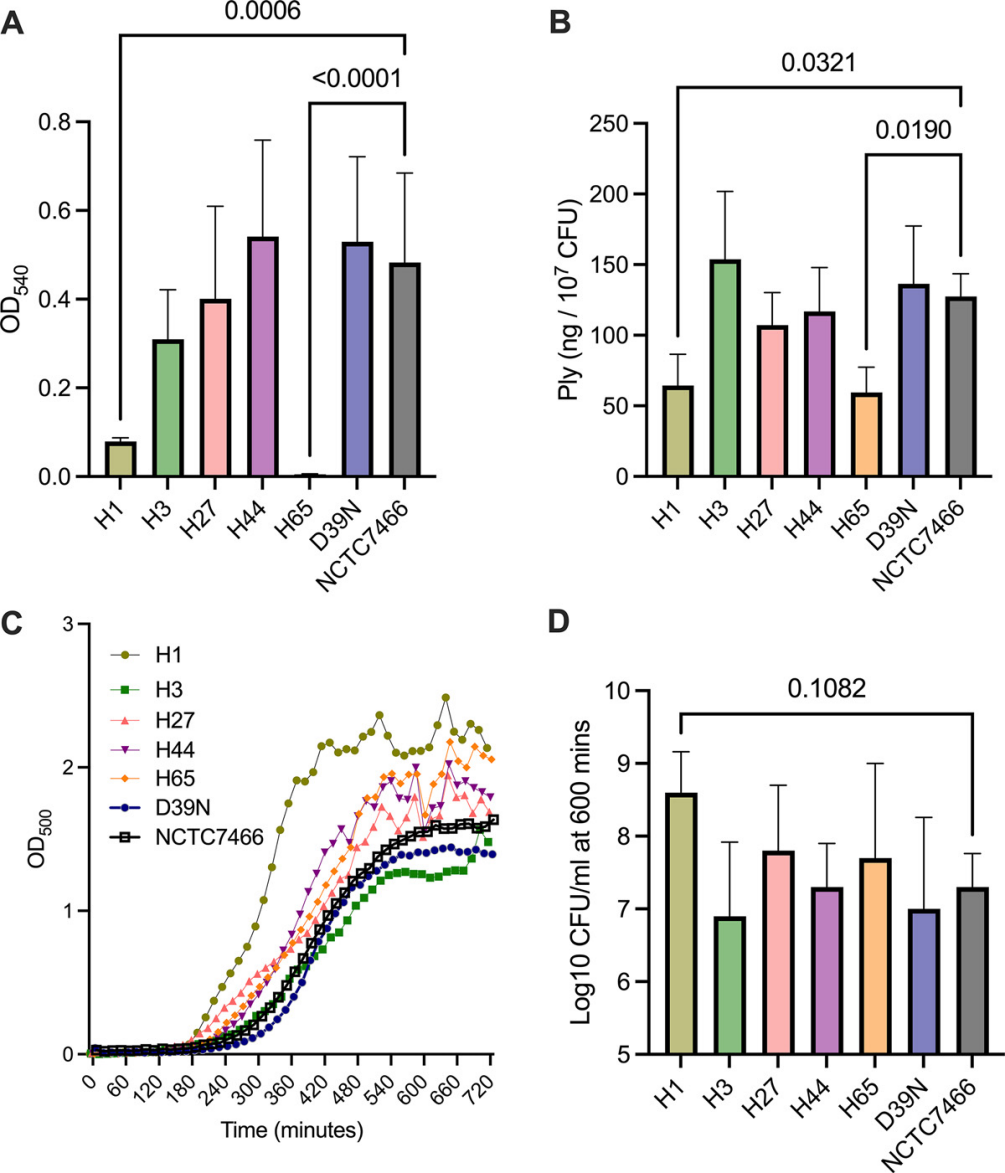
Altered virulence and growth characteristics of D39 stocks from different laboratories. (A) Hemolytic activity of D39 stocks. Bacterial lysates diluted to a total protein concentration of 5 μg were incubated with sheep red blood cells, and blood cell lysis was then determined by the absorbance at 540 nm. Data are means ± standard deviations (*n* = 5 biological replicates per group). (B) Pneumolysin concentration expressed as nanograms per 10^7^ CFU. Bacterial cell pellets were lysed and used for the detection of pneumolysin protein by ELISAs. Data are means ± standard deviations (*n* = 3 biological replicates per group). (C) Growth of D39 stocks in brain heart infusion broth. The optical density at 500 nm was measured every 15 min for 12 h. Only strain H1 demonstrated significantly different growth characteristics compared to NCTC7466 (*P* < 0.01 between 315 and 615 min by two-way analysis of variance [ANOVA] with Dunnett’s multiple-comparison test). Data are pooled from three independent experiments, for each of which the mean from two technical replicates per strain was taken. (D) The pneumococcal cell density at 10 h was determined by serial dilution onto blood agar (*n* = 4 biological replicates per group). *P* values in panels A, B, and D were determined by one-way ANOVA with Dunnett’s multiple-comparison test versus NCTC7466.

Mutations in genes implicated in virulence or antimicrobial resistance were identified in several D39 strains. H65 had acquired two SNPs in *ply* (L445S and V468E), encoding the cholesterol-dependent cytolysin pneumolysin. Both SNPs were in the region of the gene encoding domain 4 of the toxin, which is responsible for membrane binding, but not in residues previously identified as conferring hemolytic activity (19). A comparison of the hemolytic activities of the six D39 strains revealed that H65 had no detectable activity (Fig. 3A), suggesting that the two amino acid substitutions compromise protein function. The pneumolysin activities of H3, H27, H44, D39N, and NCTC7466 were comparable. H1 had significantly reduced hemolytic activity relative to all strains except H65. Both H1 and H65 retained the ability to produce pneumolysin protein, albeit at reduced levels relative to those of the other D39 stocks (Fig. 3B). No mutations were detected in or proximal to the *ply* promoter region.

Both D39N and H3 contain an SNP in *cbpD* (H39Y), encoding choline-binding protein D, a murein hydrolase involved in fratricide (20). H65 harbors an SNP in *pbp1a* (A109V), sitting within the glycosyltransfer domain of the penicillin-binding protein that is responsible for the polymerization of *N*-acetylglucosamine and *N*-acetylmuramic acid chains. The SNP is distant from the mutational hot spot in *pbp1a* associated with β-lactam resistance (21), and the susceptibility of H65 to penicillin, ampicillin, and cefotaxime was not altered relative to those of the other D39 stocks (Table S1). D39N, H3, and H27 harbor mutations in genes of the competence system, although they all display transformation efficiencies comparable to that of NCTC7466 (Table S2).

To determine the collective impact of the identified mutations, we profiled D39 growth in nutrient broth (Fig. 3C). The growth characteristics were comparable across all strains, with the exception of H1, which reached exponential phase more rapidly (*P* < 0.01 versus NCTC7466) (between 315 and 615 min). The maximum population densities achieved following 10 h of growth were comparable for all strains (Fig. 3D). Of note, all four fixed nonsynonymous SNPs that are unique to H1 are within genes encoding proteins involved in nutrient acquisition or metabolism (Table 1).

**TABLE 1:** Nonsynonymous mutations in D39 stocks from different laboratories^*a*^

Position	Mutation annotation	Gene	Description	Presence of mutation in stock					
				D39N	H3	H65	H27	H1	H44
657974	S181L	*spxB*	Pyruvate oxidase						
157206	F115L	DQM66_RS00850	M20/M25/M40 family metallo-hydrolase						
306651	Y32D	DQM66_RS01685	ATP-dependent Clp protease ATP-binding subunit						
362142	N133D	DQM66_RS01950	PTS mannitol-specific transporter subunit IIBC						
457827	Y444H	*glnA*	Type I glutamate-ammonia ligase						
500654	V323L	DQM66_RS02660	Valine-tRNA ligase						
500656	V323L	DQM66_RS02660	Valine-tRNA ligase						
837501	A260V	DQM66_RS04395	LysR family transcriptional regulator						
1019890	Y91C	DQM66_RS11435	Hypothetical protein						
1090952	P274T	DQM66_RS05705	Polyprenyl synthetase family protein						
1166597	G237V	DQM66_RS06040	Carbamoyl phosphate synthase small subunit						
1181971	A102S	*crcB*	Fluoride efflux transporter CrcB						
1234414	A398G	*aroA*	3-Phosphoshikimate 1-carboxyvinyltransferase						
1353455	L274S	*atpA*	F_o_F_1_ ATP synthase subunit alpha						
1366359	N256S	DQM66_RS07185	DEAD/DEAH box helicase						
1387760	D98G	DQM66_RS07305	NAD(P)H-dependent oxidoreductase						
1511664	P296A	DQM66_RS07940	ABC transporter substrate-binding protein						
1565733	V114F	*pknB*	Stk1 family PASTA domain-containing Ser/Thr kinase						
1607893	G53D	DQM66_RS08475	Asp23/Gls24 family envelope stress response protein						
1735190	V119A	DQM66_RS09265	Competence/damage-inducible protein A						
1744924	S115G	DQM66_RS09325	M50 family metallopeptidase						
1961957	A858V	DQM66_RS10510	Discoidin domain-containing protein						
1978062	L293P	*dltD*	d-Alanyl-lipoteichoic acid biosynthesis protein DltD						
2008202	H39Y	*cbpD*	Choline binding-anchored murein hydrolase CbpD						
243327	Y216H	DQM66_RS01355	LLM class flavin-dependent oxidoreductase						
247387	D72G	*spuA*	LPXTG-anchored pullulanase SpuA						
248184	K56T	*rpsL*	30S ribosomal protein S12						
250241	I299T	*fusA*	Elongation factor G						
250522	A393S	*fusA*	Elongation factor G						
319145	A454V	DQM66_RS01735	Sugar transferase						
342683	A109V	*pbp1a*	Penicillin-binding protein PBP1A						
638380	I218V	DQM66_RS03315	Amino acid ABC transporter ATP-binding protein						
735779	V86I	DQM66_RS03875	Amino acid ABC transporter ATP-binding protein						
753387	G437D	DQM66_RS03985	ABC transporter ATP-binding protein						
962360	R85C	*rpmA*	50S ribosomal protein L27						
1632521	T232A	*pfbA*	Multiligand-binding adhesin PfbA						
1721928	V468E	*ply*	Cholesterol-dependent cytolysin pneumolysin						
1721997	L445S	*ply*	Cholesterol-dependent cytolysin pneumolysin						
1724756	S23I	DQM66_RS09195	DUF4231 domain-containing protein						
44870	S268T	*comB*	Competence pheromone export protein ComB						
54032	Q20K	*purH*	Bifunctional purine biosynthesis protein PurH						
202272	K65T	*rplE*	50S ribosomal protein L5						
290722	A303V	DQM66_RS01595	Sugar kinase						
437741	F144L	DQM66_RS02330	ECF-type riboflavin transport substrate binding						
805481	Q501K	DQM66_RS04210	DNA polymerase III subunit alpha						
1194993	Q632*	DQM66_RS06230	ABC transporter ATP-binding protein						
1676827	A408D	*treP*	PTS trehalose-specific EIIBC component						
1681870	Q206*	DQM66_RS08890	ABC transporter ATP-binding protein						
1781250	W334C	DQM66_RS09500	Bacteriocin-associated integral membrane protein						
215632	E271D	DQM66_RS01235	ABC transporter ATP-binding protein						
318872	P363L	DQM66_RS01735	Sugar transferase						
643097	N49D	*lctO*	l-Lactate oxidase						
1677743	Q103*	*treP*	PTS trehalose-specific EIIBC component						
385012	M129I	DQM66_RS02050	MarR family transcriptional regulator						
1030176	Q286R	DQM66_RS05385	Recombinase family protein						
1466223	P110S	DQM66_RS07705	Metallophosphoesterase family protein						

a Variant calls were conducted with Breseq using NCTC7466 as the reference. The mutation position is relative to the origin of replication. The mutation annotation column indicates the original amino acid, the position within the protein sequence, and the new amino acid. Shading indicates the presence of the given mutation. PTS, phosphotransferase system. * denotes nonsense mutations resulting in introduction of premature stop codons. LLM is luciferase-like monooxygenase. ECF is energy-coupling factor.

**FIG 4:**
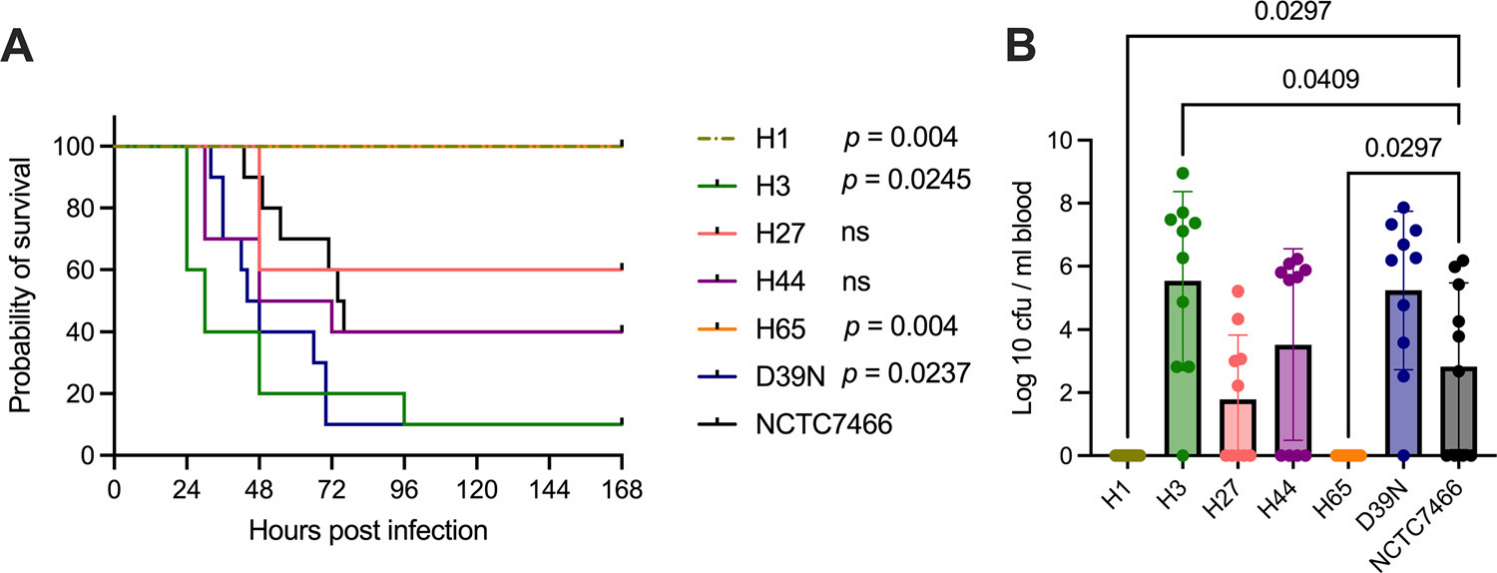
D39 virulence in a mouse invasive pneumonia model. Mice were infected intranasally with 1 × 10^6^ CFU of D39 in 50 μL of PBS. (A) Survival of 10 mice per group. *P* values were determined by a log rank test versus NCTC7466, with adjustment for multiple comparisons. ns, nonsignificant. (B) Blood bacterial burdens at 24 h postinfection, determined by tail vein blood draws. *P* values were determined by one-way ANOVA with Dunnett’s multiple-comparison test versus NCTC7466.

Next, D39 virulence was compared in a mouse pneumonia model. D39N and H3, between which there are no fixed nonsynonymous SNP differences, were the most virulent, with 90% mortality being observed within 4 days of infection (Fig. 4A). H1 and H65, the two D39 stocks that showed reduced hemolytic activity (Fig. 3A), were completely virulence attenuated, with all mice surviving the full 7 days of the experiment and no evidence of bacteremia at 24 h postinfection (Fig. 4B). H27, H44, and NCTC7466 showed intermediate virulence characteristics (40 to 70% mortality) (Fig. 4A and B). Thus, mutations acquired during simple laboratory handling procedures can lead to cumulative changes that can profoundly influence strain virulence.

### Protocols used to standardize virulence in pneumococci alter bacterial population structure.

**FIG 5:**
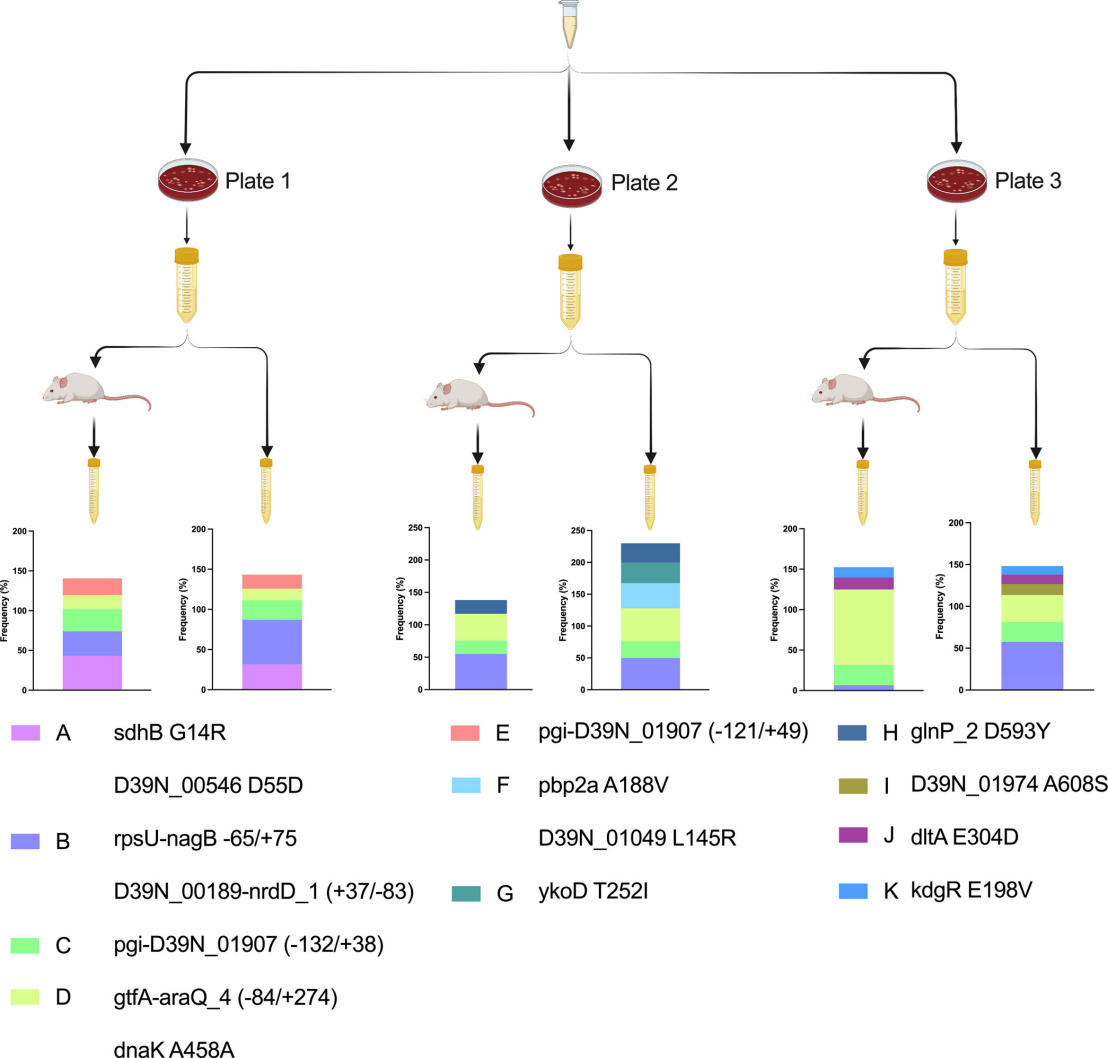
Changes in the D39 population structure during culture or mouse passage. Three blood agar plates were prepared from a single freezer stock of D39N. From each plate, a single culture was grown overnight in brain heart infusion (BHI) broth. Cultures grown overnight were split, with one portion being used to infect a mouse via intraperitoneal administration and the other portion being used for subculture into fresh BHI broth. Bacteria were recovered via cardiac puncture of mice at 8 h postinfection (passaged stocks) or at mid-exponential phase in BHI broth (nonpassaged stocks). The D39 population structure was determined from Illumina sequencing data using Breseq, with a D39N consensus sequence derived from the same freezer stocks as the reference. The identified genotypes are color-coded and are shown in the key, along with the single-letter identifier and a description of the 1 to 2 variants that define them. Stacked histogram plots depict variant frequencies. Genotype frequencies add up to more than 100%, as some genotypes sit within (i.e., are descended from) others.

Animal models of pneumococcal infection are widely used, but there is little standardization between laboratories. One approach has been to passage bacteria through mouse models, to normalize virulence, prior to use in further infection studies. This ensures batch-to-batch consistency in infection stocks, but its influence on bacterial genetics has not been determined. We used D39N to prepare three independently passaged sets of infection stocks according to previously described protocols (9). High-density pneumococcal cultures were administered to mice by intraperitoneal injection. For this, we used the three cultures grown overnight that had been prepared for the first part of this study (Fig. 1), which were derived from three individual ceramic beads from the same freezer stock tube. Bacteria recovered from the blood of mice at 8 h postinfection were then cultured in nutrient broth until mid-exponential phase, before aliquoting into single-use tubes. In parallel, we prepared three sets of nonpassaged stocks from the same starting cultures as those used for intraperitoneal injection (Fig. 5). Thus, all of the D39N stocks described in this study were derived from the same three ceramic beads from our frozen stocks.

We analyzed the population structures of D39 in passaged and nonpassaged stocks, considering both synonymous and nonsynonymous SNPs, including those that fell in intergenic regions. The factor exerting the greatest influence on population structure was the initial process of the recovery of frozen bacteria on agar and growth in nutrient broth (Fig. 5). Few genotypes were shared between stocks from separate agar plates despite the use of a single freezer stock for their preparation. Of the 11 most abundant genotypes, defined by Breseq (22, 23), across the three passaged and three nonpassaged stocks, only 3 (genotypes B, C, and D in Fig. 5) were common to stocks prepared from each of the three agar plates. In contrast, passaged and nonpassaged stocks prepared from the same agar plate shared multiple genotypes. All five genotypes in nonpassaged stocks from plate 1 were also identified in the corresponding passaged stocks. For plates 2 and 3, 4/6 and 5/6 genotypes, respectively, were shared between passaged and nonpassaged stocks (Fig. 5).

**FIG 6:**
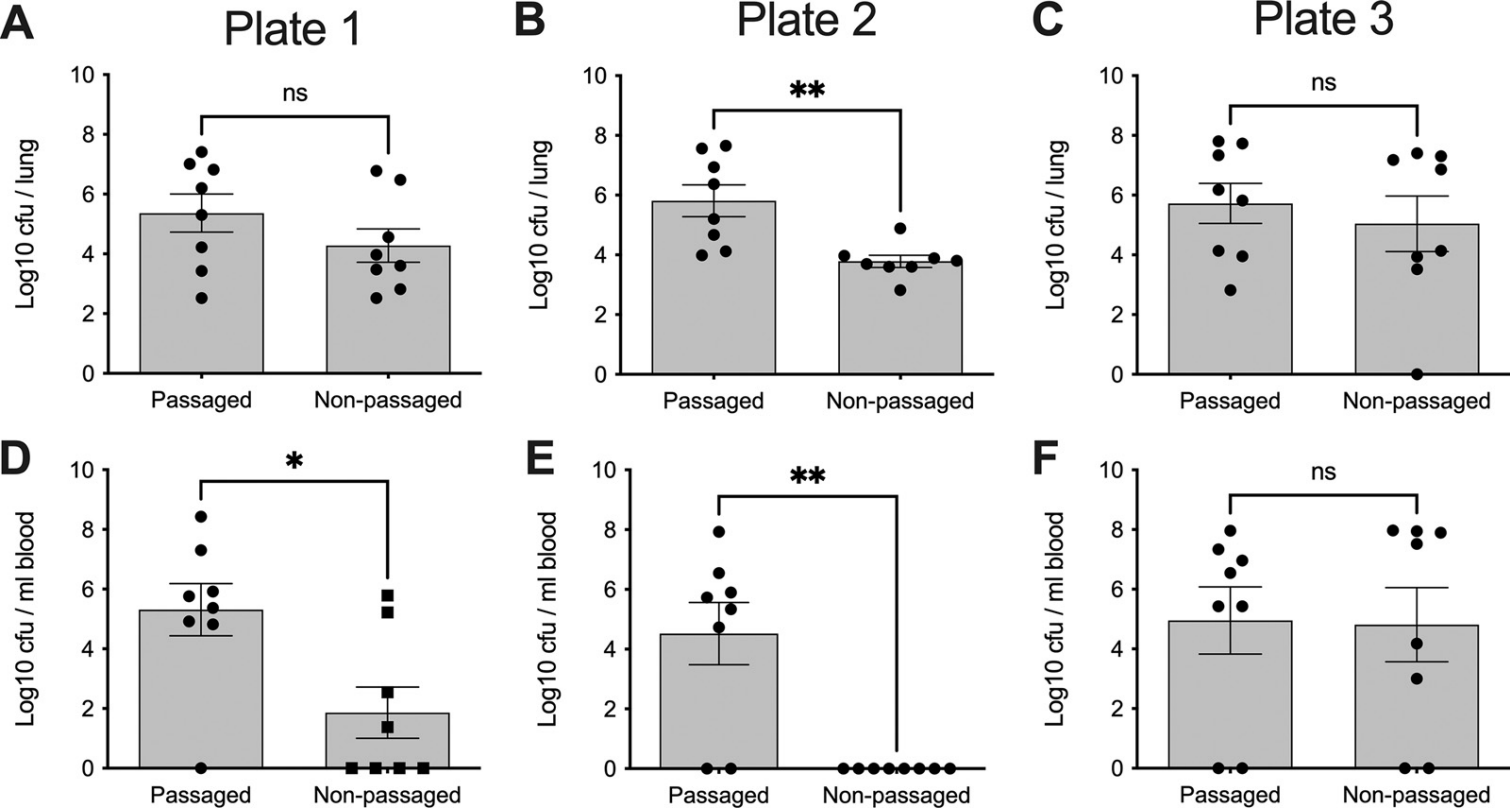
Comparative virulence of three passaged and three nonpassaged D39 cultures prepared from a single freezer stock. Mice were infected intranasally with 1 × 10^6^ CFU in 50 μL of PBS. Lung (A to C) and blood (D to F) bacterial burdens were determined at 24 h postinfection. Data are from a single experiment using cultures prepared from agar plate 1 (A and D), agar plate 2 (B and E), or agar plate 3 (C and F), as depicted in Fig. 5. *, *P* < 0.05; **, *P* < 0.01; ns, nonsignificant (by an unpaired two-tailed *t* test).

Despite the genetic similarity of passaged and nonpassaged stocks prepared from the same plate, the virulence characteristics were distinct in a pneumonia infection model (Fig. 6). Passage achieved virulence standardization, with passaged stocks from all three plates establishing high-density lung infection and a high incidence of bacteremia in mice. In contrast, nonpassaged stocks showed lower virulence, with the successful establishment of lung infection but a lower incidence of bacteremia and greater variance in outcomes between stocks prepared from different agar plates. Only nonpassaged stocks from agar plate 3 showed virulence comparable to that of the passaged stocks (Fig. 6).

### Selection of rare, high-fitness genotypes during passage.

**FIG 7:**
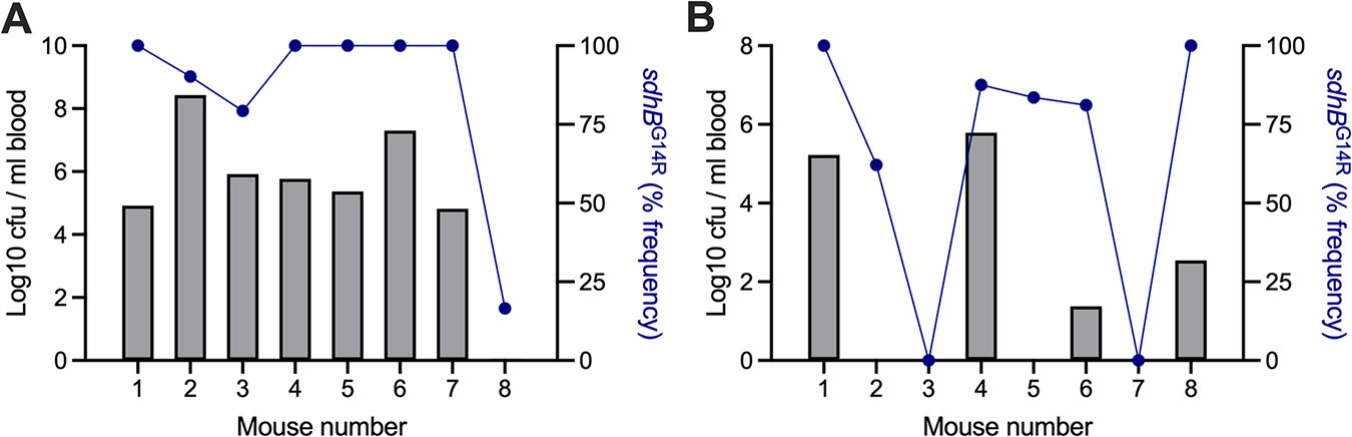
Success of the D39 *sdhB*^G14R^ genotype during invasive pneumonia in mice. Mice were infected intranasally with 1 × 10^6^ CFU of either passaged (A) or nonpassaged (B) stocks of D39N prepared from agar plate 1, as depicted in Fig. 5. Graphs show the bacterial density in the blood at 24 h postinfection (gray bars on the left *y* axis) and the frequency of the *sdhB*^G14R^ genotype in the total recovered pneumococci at 24 h postinfection (blue lines on the right *y* axis). The total recovered pneumococci are those isolated from the lungs of mice that were nonbacteremic at the time of death or those pooled from the lungs and blood of mice that were bacteremic at the time of death.

Passaged and nonpassaged stocks from agar plate 1 contained the same 5 dominant genotypes but with the expansion of genotype A, at the expense of genotype B, in the passaged stocks. Genotype A is defined by nonsynonymous mutations in *sdhB* (G14R) and a synonymous SNP in *D39N_00546*. In agar plate 1 stocks, these mutations are also linked to a nonsynonymous SNP in *D39N_01974* (A608S). The *D39N_01974* mutation, but not the genotype A mutations, was present in our freezer stocks and in stocks prepared from agar plates 2 and 3. It appears, therefore, that on agar plate 1, genotype A arose within or was expanded from the proportion of the freezer stocks harboring the *D39N_01974* mutation. To determine whether the expansion of this genotype in passaged stocks might account for their enhanced virulence relative to that of the nonpassaged stocks, we analyzed the population structure of pneumococci recovered from mice infected as part of the virulence testing performed for Fig. 6. Of the D39 populations recovered from the eight mice infected with agar plate 1 passaged stocks, seven showed evidence of a further increase in the frequency of genotype A, with the *sdhB*^G14R^ mutation becoming fixed in bacterial populations recovered from five mice (Fig. 7A). The one mouse in which no bacteremia was evident at 24 h postinfection was the only one harboring a D39 population in the lungs that had a decreased genotype A frequency relative to those of the stocks used for infection (Fig. 7A).

In mice infected with agar plate 1 nonpassaged stocks, the severity of infection was positively correlated with the *sdhB*^G14R^ mutation frequency in the bacterial population. The 4/8 mice that developed bacteremia all had D39 populations with a >75% frequency of the mutation, while of the 4 mice that did not develop bacteremia, 2 had D39 that had lost the *sdhB*^G14R^ variant altogether (Fig. 7B). Thus, the passage of agar plate 1 stocks achieved virulence standardization by enriching for a genotype associated with enhanced *in vivo* fitness. The passage process did not fix the genotype in the infection stocks (Fig. 5) but, by enabling its expansion, increased its chances of passing successfully through population bottlenecks in subsequent *in vivo* experiments (Fig. 6). Thus, a rare genotype at an undetectable frequency (<2%) in our D39N freezer stocks was enriched during passage due to the advantages that it confers within the host.

### Loss of genotypes during passage.

Passaged and nonpassaged stocks prepared from agar plate 2 behaved comparably to those prepared from plate 1 (Fig. 6) despite their very different population structures (Fig. 5). Plate 2 nonpassaged stocks shared genotypes B, C, and D with stocks from plate 1 but were additionally characterized by genotypes F, G, and H. Of those, genotypes F and G were not observed in the passaged stocks prepared from the same plate. The four shared genotypes (B, C, D, and H) were found at comparable frequencies in the passaged and nonpassaged stocks (within 10%), suggesting that the loss of genotypes F and G during passage might explain the increased ability to establish and maintain bloodstream infection in passaged stocks. Genotype F (39.5% frequency in nonpassaged stocks) is defined by two nonsynonymous SNPs, in *pbp2a* (A188V), encoding a cell wall synthase, and a gene encoding a hypothetical protein (*D39N_01049* L145R). Genotype G (32.3% frequency) is defined by a nonsynonymous SNP in *ykoD* (T252I), encoding an hydroxymethylpyrimidine HMP/thiamine import ATP-binding protein. The passage of agar plate 1 stocks did not lead to genotype loss or gain but enriched for previously rare, high-fitness variants. In contrast, the passage of agar plate 2 stocks resulted in the elimination of previously high-frequency, low-fitness variants. The two scenarios resulted in comparable stock virulence potentials by increasing the chances of suitably fit genotypes passing through population bottlenecks.

### Coexistence of genotypes with comparable *in vivo* fitness.

**FIG 8:**
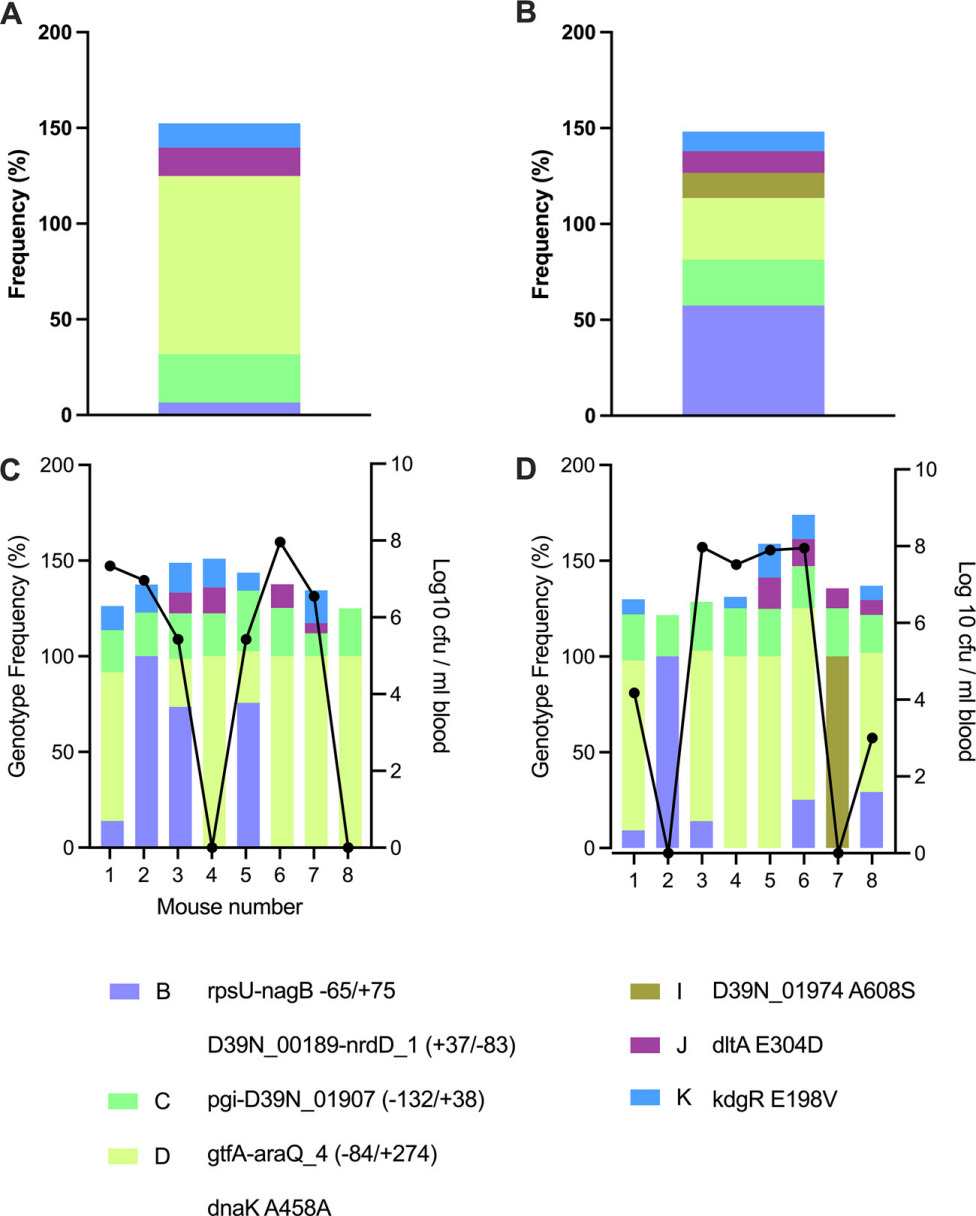
Relationship between genotype frequency and virulence in D39 prepared from agar plate 3. (A and B) The frequencies of genotypes in passaged (A) and nonpassaged (B) stocks of D39N prepared from agar plate 3 are shown in stacked histogram plots. Each color represents a different genotype, as defined in the key. (C and D) Mice were infected intranasally with 1 × 10^6^ CFU of D39N from passaged (C) or nonpassaged (D) stocks prepared from agar plate 3. The blood and lungs were collected at 24 h postinfection, and plots depict the genotype frequencies in the total recovered pneumococci from individual mice (stacked histograms on the left *y* axis) and the bacterial density in the blood (black lines on the right *y* axis). The total recovered pneumococci are those isolated from the lungs of mice that were nonbacteremic at the time of death or those pooled from the lungs and blood of mice that were bacteremic at the time of death.

Plate 3 passaged and nonpassaged stocks shared 5 of 6 genotypes, with only genotype I, defined by the same nonsynonymous mutation in *D39N_01974* that was linked to genotype A in agar plate 1 stocks, being lost during passage. The virulence of plate 3 nonpassaged stocks was higher than the virulence of those from plates 1 and 2 (Fig. 6). The *in vivo* fitness of the genotypes present in both the passaged and nonpassaged stocks from agar plate 3 is apparent from the observation that all input genotypes were still present in pneumococcal populations recovered from at least one mouse after virulence testing (Fig. 8A to D). However, genotype I, which was lost during passage (Fig. 8A), was also lost during virulence testing in 7 of 8 mice infected with the nonpassaged stocks (Fig. 8D).

Collectively, these data demonstrate that modest shifts in the frequency of rare genotypes in bacterial stocks can impact experimental outcomes in the short term and drive within-strain diversification in the long term. These shifts arise both from genetic drift during population bottlenecks that are inherent within culture protocols and from selection during *in vitro* and *in vivo* passage.

## DISCUSSION

The short generation time of many bacterial pathogens, at least under laboratory conditions, presents researchers with both an opportunity and a challenge. High-density cultures can be prepared in a matter of hours for experiments. Adaptive evolutionary processes can be studied over months, weeks, or even days. Genetically modified strains can be generated rapidly and characterized. However, as a research community, we have given less consideration to how these features of bacterial biology, which enable their study in the laboratory, might also confound our experimental observations. Those working with bacterial pathogens will be aware that within-strain genotypic and phenotypic differences exist and have been documented (1). However, there has been no coordinated effort to introduce standardized bacterial handling practices that might minimize the influence of genetic drift or selection on strain divergence in laboratory settings. Such practices would improve the reproducibility of findings across different spatial and temporal settings.

We sought to characterize how short-term culture of Streptococcus pneumoniae might influence the population structure of a single strain, D39. We had previously identified more than 30 low-frequency genotypes within our long-term freezer stocks of D39 (2) and reasoned that some of these might influence experimental outcomes, either through the selection of rare but beneficial genotypes or where low-fitness genotypes become randomly enriched following population bottlenecks. Our findings here suggest that both of these factors are contributory. We observed the enrichment or elimination of genotypes present in our original freezer stock during culture as well as the acquisition of new mutations. More than 75% of the mutations that we observed in D39 cultures were not identified in our freezer stocks, although a proportion of these may have been present at undetectable frequencies.

The process of passaging pathogens through animal infection models has been widely used (3–9). For viruses, if no suitable cell culture model for virion expansion exists, animal passage may be essential. For bacterial pathogens, the process is not required but may be desirable, as it is thought to both select for and standardize virulence. Our findings suggest that passage achieves both of these objectives but that, in doing so, it significantly alters strain genetics. The clearest example of this was in the stocks that we prepared from agar plate 1 (Fig. 5 to 7). Passage enriched a previously rare genotype, characterized by a nonsynonymous mutation in *sdhB*. Although this genotype accounted for just over one-half of the total D39 population (~52%) following passage, it exerted a disproportionate influence on subsequent experimental outcomes. We challenged 8 mice with the passaged stocks (*sdhB*^G14R^ at 52%) and another 8 mice with nonpassaged stocks prepared from the same plate (*sdhB*^G14R^ at 31%). By 24 h postinfection, 11 of 16 mice had developed bacteremia. Sequencing of the D39 population recovered from the mice at 24 h postinfection demonstrated that the *sdhB* genotype frequency had increased during each of these infections, becoming fixed in 7/11 mice. In contrast, in the 5 nonbacteremic mice, sequencing of pneumococci recovered from the lungs revealed that the *sdhB* genotype had decreased in frequency in 3/5 cases. The fate of the *sdhB* genotype over the course of the infection was thus a strong determinant of the severity of that infection. Of note, *sdhB* has previously been identified as being essential for the establishment of lung infection by pneumococci (24).

These observations have implications for how we think about minority variants in bacterial stocks. We were aware of the *sdhB*^G14R^ genotype in our infection stocks only as we had undertaken population-level sequencing. A consensus sequence for our strain would not have included it, and we did not identify it by population-level sequencing of our original freezer stocks of D39 or stocks prepared from agar plates 2 and 3. Similarly, we did not identify the presence of the mutation in pneumococci isolated directly from agar plate 1, although it was present following liquid culture overnight and was maintained in both the passaged and nonpassaged stocks. However, we identified an identical *sdhB*^G14R^ variant in a previous experimental evolution study, where it was observed in 2 of 10 D39 lineages that had been serially passaged through a mouse pneumonia model (2). The identification of the same mutation in that study and this one suggests that the genotype must be present at a low frequency in our freezer stocks. We sequenced these stocks to a depth sufficient to identify any variant at a ≥2% frequency and did not detect the *sdhB* mutation, highlighting that even very rare variants can substantially impact experimental outcomes if they confer a sufficiently strong fitness advantage.

The benefits of population-level sequencing of bacteria have been well documented by others (25), but it is notable here that the differences in *in vivo* virulence between different stock preparations derived from a single freezer aliquot of D39 are of a magnitude similar to those observed when comparing wild-type and gene deletion mutant strains in experiments designed to identify virulence factors. Confidence in experimental data from *in vivo* infection models is thus predicated on understanding the population structures of the bacterial input populations.

The process of preparing passaged bacterial stocks involves several steps at which population bottlenecks and changing selective pressures might influence the genetic architecture of the bacterial culture. Although the *in vivo* step of the protocol is presumed to be the principal point of selection, subsequent growth in liquid culture, inoculated with blood retrieved from infected mice, might also contribute. Blood is collected from mice 8 h after intraperitoneal infection, at which point the animals display visible signs of sepsis, including reduced energy, hunching, and piloerection of the fur. However, the number of pneumococci in the blood is not typically quantified at this step of the protocol, and there is the potential for mouse-to-mouse variations in bacterial numbers and, thus, the size of the population bottleneck introduced during blood sampling. This may in turn influence the genetic makeup of the resulting bacterial stocks and also compromise the ability to define that genetic makeup if there is a high proportion of dead bacteria from overgrown cultures.

It should be noted that the experiments described here were designed to reflect commonly practiced microbial culture techniques rather than to minimize the impact of culture on the genetic structure of the bacterial population. There is scope for refining these procedures to reduce the impacts of bottlenecks and selection bias. Sampling single colonies from plates, rather than sweeps, would provide a more homogeneous starting culture, but the choice of the colony would heavily influence the genetic structure of the resulting stocks given the possibility of selecting a colony derived from a previously rare genotype. Conversely, collecting the totality of the growth on agar would produce a starting culture more reflective of the genetic composition of the freezer stocks, increasing reproducibility between experiments, provided that the genetic structure of the original freezer stocks is understood. Variability arising during liquid culture could be reduced by minimizing incubation times and avoiding the use of late-exponential-phase and stationary-phase cultures, where cell death might introduce bottlenecks and genetic exchange via transformation may contribute to genetic diversification. These considerations must, however, be balanced against the need to generate high-density cultures for experimental use as well as the practicalities for the researcher. Culture overnight is common because it allows the propagation of bacterial stocks within working hours. Achieving mid-exponential-phase cultures directly from sampling growth on agar may be more desirable but can take upwards of 12 h, with timings being less predictable than those observed following the subculture of cultures grown overnight in broth.

In the comparison of the six D39 stocks acquired from different laboratories and in the studies of variation arising during culture and animal passage, mutations in genes of the competence system were frequently identified. Such mutations may have contributed to the gains and losses of diversity observed, by enhancing or restricting genetic exchange between coexisting genotypes. Although those contributions were not directly assessed in the present study, others have highlighted transformation as the primary driver of pneumococcal evolution (26–28).

The long-term consequence of fluctuations in the genotype frequency during laboratory handling of S. pneumoniae is that some variants will become fixed, either by chance or under selection. In the most striking example here, we observed the loss of activity of the pneumolysin toxin, a crucial virulence determinant in pneumococci (29), in one of the D39 stocks from our collection. For widely used reference strains like D39, this gradual within-strain divergence is problematic as such strains are often benchmarks or controls in comparative experiments.

Collectively, these data highlight the need for careful and standardized handling of bacterial pathogens in laboratory settings. Population-level sequencing is a valuable tool to aid in understanding the contributions that minority variants make to pathogen biology. Short-read sequencing costs have dropped rapidly over the last decade, and there are a growing number of bespoke microbial sequencing services, making technology and expertise affordable and accessible to most research groups. At a minimum, we advocate for the periodic sampling and sequencing of long-term freezer stocks from culture collections, particularly during the onward propagation of strains or for extensively used stocks that are exposed to repeated freeze-thaw cycles. It may also be of value to sequence input and output bacterial populations from experimental assays. Sharing of culture collection stocks between laboratories at the onset of new collaborative projects would minimize the impact of interlaboratory, within-strain diversity on experimental outcomes.

## MATERIALS AND METHODS

### Ethics statement.

This study was performed in accordance with United Kingdom Home Office guidelines (30) under project license PP2072053. Animal experiments were performed at the University of Liverpool with approval from the University of Liverpool animal welfare and ethical review board (AWERB). Mice were purchased from Charles River, United Kingdom.

### Bacteria.

**(i) Generation of nonpassaged stocks.** Culture steps were performed at 37°C with 5% CO_2_. Pneumococci from a frozen aliquot of ceramic beads (31) (Mast Cryobank) were cultured overnight on blood agar base (BAB) plates containing 5% (vol/vol) horse blood. Approximately 20 colonies were then inoculated into brain heart infusion (BHI) broth (Oxoid, UK) and cultured overnight to early stationary phase (~14 h; optical density at 500 nm [OD_500_] of 1.6 to 1.8). The remaining growth on the agar plate was collected for whole-genome sequencing (tabs labeled “Plate” in Data Sets S1 to S3 in the supplemental material). A portion of the culture grown overnight in broth was subcultured into fresh BHI broth supplemented with 20% (vol/vol) heat-inactivated fetal bovine serum (FBS) to achieve an OD_500_ of ~0.5. The remaining culture grown overnight was retained for the generation of passaged stocks and whole-genome sequencing. Subcultures were incubated statically for 5 h (until mid-exponential phase; OD_500_ of ~1.0), frozen in 500-μL aliquots in cryovial tubes, and stored at −70°C.

**(ii) Generation of passaged stocks.** The remaining volume of cultures grown overnight that were used for the preparation of nonpassaged stocks was centrifuged to pellet the bacteria. The bacterial pellet was resuspended in 5 mL 1× phosphate-buffered saline (PBS) to give an OD_500_ of 1.2 to 1.4. Using a 0.5-mL insulin syringe, 100 μL of the pneumococcal suspension (~1 × 10^7^ CFU/mouse) was intraperitoneally (i.p.) injected into 7- to 8-week-old female CD1 mice. Eight hours after i.p. injections, by which time the mice showed evidence of sepsis (reduced movement, hunching, and piloerection of fur), mice were culled, and 300 to 500 μL of blood was removed by cardiac puncture. The blood was added to BHI broth and incubated statically for 16 to 20 h. The bacterial culture was carefully removed, without disturbing the pellet of red blood cells (RBCs) at the bottom of the tube, and centrifuged at 3,000 rpm for 15 min. Bacteria were resuspended in BHI broth containing 20% (vol/vol) FBS. Cultures were incubated statically for 5 h (until mid-exponential phase; OD_500_ of ~1.0). Cultures were frozen in 500-μL aliquots in cryovial tubes and stored at −70°C.

### DNA extractions.

Genomic DNA was extracted from fresh cultures of D39 at different stages of growth or after different laboratory handling steps, as described in Results. As a prelysis step, a pellet from 2 mL of a culture grown overnight was incubated for 1 h at 37°C with lysozyme (Sigma) and mutanolysin (Sigma) in Tris-EDTA (TE) buffer. Proteinase K (Qiagen) and buffer AL (Qiagen) were then added, and the samples were incubated at 56°C for 2 h. The DNeasy blood and tissue kit (Qiagen) was used according to the manufacturer’s instructions for the purification of DNA from Gram-positive bacteria. DNA quality was assessed by the absorbance ratios at 260/280 nm and 260/230 nm using a NanoDrop spectrophotometer (Thermo Fisher Scientific); ratios of ≥1.8 were used as an indication of quality DNA. A Qubit fluorometer system with a broad-range DNA kit (Invitrogen) was used to quantify DNA. For Illumina sequencing, >1 μg of DNA at a concentration between 20 and 150 ng/μL was provided.

### Illumina sequencing and read processing.

Short-read sequencing was performed to produce 151-bp paired-end reads on an Illumina HiSeq 2500 platform at The Wellcome Trust Sanger Institute, United Kingdom. Analysis of sequencing data was carried out using a virtual machine hosted by the Cloud Infrastructure for Microbial Bioinformatics (CLIMB) consortium (32). Raw Illumina reads were processed using Trim Galore (v0.4.4) with Cutadapt (v1.9.1) for paired-end reads, using default settings, to remove short low-quality reads (<20 bp), trim Illumina adaptor sequences, and eliminate poor-quality bases from the sequences (<*Q*_20_). FastQC (v0.11.5) confirmed that the trimmed reads were of sufficient quality for analysis.

### Variant calling.

Short sequence reads of D39 stocks acquired from different laboratories (D39N, H1, H3, H27, H44, and H65), were mapped to the NCTC7466 genome sequence (BioProject accession number PRJNA872357) to identify mutations using Breseq (v0.31.1) (22). Default settings were applied, with the predict-polymorphisms function. This identified the percent frequency of variants within each D39 population in comparison to the NCTC7466 reference. Synonymous and nonsynonymous SNPs as well as insertions/deletions and intergenic mutations were detected. The short sequence reads of bacteria obtained from culture or mouse passage were mapped to the D39N genome sequence (BioProject accession number PRJNA658145) (2) to identify mutations using Breseq, as described above.

### Murine models of infection.

Mice were housed at the University of Liverpool biomedical services unit at 21°C to 23°C with 55 to 65% humidity. Mice were placed into individually ventilated cages (GM500; Tecniplast). Automatic watering provided reverse-osmosis water sterilized by UV radiation. Enrichment included nesting material, a balcony, a dome home, and a handling tunnel. For virulence testing, 7- to 8-week-old female C57BL/6 mice were used, after 7 days of acclimatization to the animal unit. Mice were anesthetized with O_2_-isoflurane and infected intranasally with 1 × 10^6^ CFU of S. pneumoniae in 50 μL of PBS. Mice were periodically scored for clinical signs of disease and culled when severity limits were reached or at predetermined times postinfection. Severity limits are based on a scoring system that considers animal appearance (hunching and changes in the coat), natural and provoked behavior (depressed or elevated activity), weight loss, and respiratory patterns. A moderate change across three or more categories, or a substantial change in any single category, is the defined severity limit. Blood samples were obtained through tail bleeds or cardiac punctures under terminal anesthesia.

### Determination of bacterial numbers in the lungs and blood.

Viable counts of pneumococci in murine whole blood and lung homogenates were determined by serial dilution in PBS and plating onto BAB plates containing 5% (vol/vol) defibrinated horse blood and 40 μg/mL of gentamicin (Sigma). Plates were incubated overnight at 37°C with 5% CO_2_, and bacterial colony numbers were assessed the following day.

### Hydrogen peroxide detection assays.

H_2_O_2_ was quantified from mid-exponential-phase cultures using the Amplex Red hydrogen peroxide/peroxidase assay kit (Thermo Fisher). Pneumococci were pelleted by centrifugation, washed with PBS, and resuspended in PBS containing 0.5 mM glucose. After 1 h of incubation at 37°C with 5% CO_2_, the supernatants were diluted in Amplex Red reaction buffer. The Amplex Red reagent (10-acetyl-3,7-dihydroxyphenoxazine) reacts with H_2_O_2_ to produce resorufin, a red-fluorescent oxidation product. The fluorescence at 585 nm was detected using a FLUOstar Omega plate reader (BMG Labtech). Raw fluorescence readings were normalized to the optical density of the bacterial input cultures. Results were expressed as a percentage of H_2_O_2_ production relative to NCTC7466.

### *In vitro* growth curves.

Pneumococci were streaked onto BAB plates and incubated overnight at 37°C with 5% CO_2_. A sweep of colonies was inoculated into 5 mL of BHI broth to obtain an OD_500_ of ≥0.05. The OD_500_ was further adjusted to 0.002, and 200 μL of the bacterial culture was added to a 96-well microplate, with 3 to 4 replicates/sample. BHI broth was used as a blank control. Plates were incubated for 12 h, with readings of the OD_500_ every 15 min using a FLUOstar Omega plate reader (BMG Labtech).

### Hemolytic assays.

A 4% RBC solution was prepared by adding 400 μL of pelleted sheep red blood cells to 10 mL of PBS. Bacterial stocks were thawed, centrifuged, and resuspended at a concentration of 8.9 × 10^7^ CFU/mL. Bacteria were centrifuged again, the supernatant was removed, and the pellet was resuspended in 150 μL of 0.1% sodium deoxycholate to lyse the bacteria. After culture for 1 h at room temperature, 25 μL of the suspension was removed for protein concentration determination using a Pierce bicinchoninic acid (BCA) protein assay kit (Thermo Fisher Scientific). Ten micrograms of protein from lysed bacteria was added in a 1:1 ratio to a 4% RBC solution in a microwell plate before being diluted 2-fold with a 4% RBC solution. After 30 min at 37°C, the plate was centrifuged, and the supernatant was carefully removed and placed into a flat-bottomed microwell plate. The OD_540_ was measured to determine the levels of hemoglobin released.

### Pneumolysin ELISA.

Enzyme-linked immunosorbent assay (ELISA) microplates (Corning Laboratories, Corning, NY) were coated overnight at 4°C with 1 μg per well of mouse anti-Ply (PLY-4) antibody (catalog number ab71810; Abcam). After washing with PBS plus 0.05% Tween, plates were blocked for 3 h with PBS plus 20% FBS. Pneumococcal lysates were prepared from cultures grown overnight that were pelleted and resuspended in PBS plus sodium deoxycholate at 10%. The lysates were pelleted, and the supernatant was used for the assay. For the standards, a 2-fold pneumolysin toxin dilution series was prepared. Bacterial lysates and standards were added to ELISA plates and incubated at room temperature for 2 h. After washing, 1 μg/well of rabbit anti-Ply antibody (catalog number ab71811; Abcam) was added, and the plates were incubated for 2 h. The plates were washed, and goat anti-rabbit alkaline phosphatase antibody (catalog number ab97048; Abcam) was added for 30 min at room temperature. After washing, 100 μL/well of p-nitrophenyl phosphate pNPP color reagent (Sigma) was added for 30 min, and the plates were incubated in the dark before the reaction was stopped with 100 μL of 1 M NaOH. The absorbance was measured at 405 nm in a Varioskan multimode microplate reader (Thermo Fisher Scientific).

### Antimicrobial susceptibility testing.

MIC values were determined according to the broth microdilution protocol outlined by CLSI guidelines for antimicrobial susceptibility testing (33). Pneumococci were grown on BAB plates overnight at 37°C with 5% CO_2_. Cultures grown overnight in BHI broth from plate sweeps were incubated for 18 h. Antibiotic susceptibility against a 2-fold serial dilution of each antibiotic, from 1 μg/mL to 0.03125 μg/mL, was tested. Each well was inoculated with 50 μL of an overnight-grown culture adjusted with BHI broth to an OD_600_ of 0.1 using a Spectronic 200 spectrophotometer (Thermo Fisher Scientific). Wells were inoculated within 30 min of standardization. Plates were incubated for 18 h, and the OD_600_ was measured using a SPECTROstar Nano instrument (BMG Labtech). The percentage of growth compared to the no-antibiotic control was calculated.

### Transformation assays.

The generation of competent D39 cells and subsequent transformation were performed using complete transformation medium (C+Y medium) methods as described previously (34). Cells were transformed with 200 ng of a PCR product carrying the *aad9* gene, conferring resistance to spectinomycin, which was amplified with primers aad9-FW (5′-GAACTAGTGGATCCCCCGTTTG-3′) and aad9-REV (5′-CAATACGGGATAATACCGCGC-3′) from plasmid pR412 (35). Transformants were selected on blood agar base medium supplemented with 400 ng/mL spectinomycin. After incubation for 24 h at 37°C, colonies that were resistant to spectinomycin were counted. The transformation efficiency was expressed as CFU per milliliter of transformed competent cells.

### Statistical analysis.

Statistical analysis was carried out using the GraphPad Prism version 9 statistical package (GraphPad Software, Inc.). Data were tested for normality, and the tests used are defined in the figure legends. *P* values were adjusted for multiple comparisons.

### Data availability.

Trimmed FastQ files of Illumina paired-end sequence reads are available from the NCBI (BioProject accession number PRJNA872357).
