# Supplementary material for: Influence of Streptococcus pneumoniae Within-Strain Population Diversity on Virulence and Pathogenesis
Source: Microbiol Spectr. 2022 Dec 12;11(1):e03103-22. doi: 10.1128/spectrum.03103-22 (PMC9927508; doi:10.1128/spectrum.03103-22)
Supplement: Supplemental file 5 — Supplemental material. Download spectrum.03103-22-s0001.pdf, PDF file, 0.1 MB [file spectrum.03103-22-s0001.pdf]

Supplementary Information

Supplementary Figure 1. Hydrogen peroxide production by six D39 strains. Data are presented as relative (percentage) H_2O_2 production for each strain versus NCTC7466, after normalisation for cell density, as determined by Amplex Red Hydrogen Peroxide detection kit. Data are from two independent experiments. Statistical analysis was by ordinary one-way ANOVA with Dunnett's multiple comparison test vs NCTC7466. Only statistically significant differences are shown.

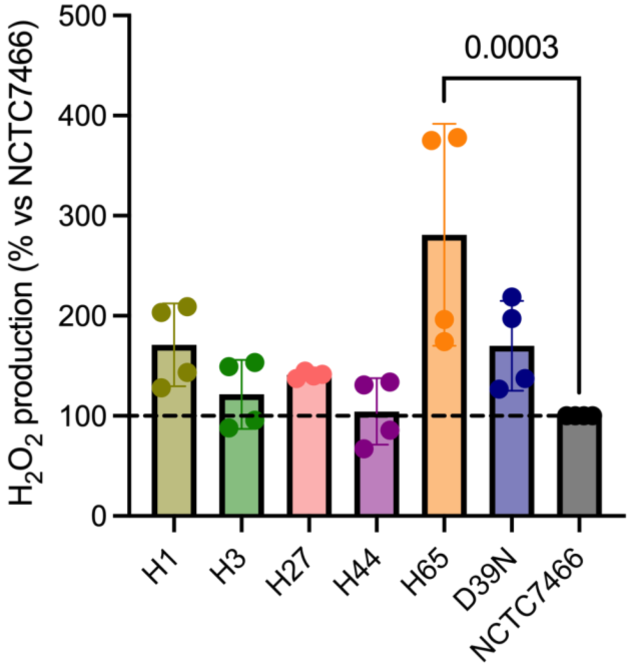

Supplementary Table 1. Minimum inhibitory concentrations of beta lactam antibiotics against six D39 strains. Data are presented as median MIC50 (μg/ml) with the range of observed MIC50 values from three independent experiments, each containing three technical replicates per strain.

Strain	Cefotaxime	Penicillin	Ampicillin
H1	0.0039 (0.0039-0.0078)	0.0039 (0.0039-0.0078)	0.1 (0.06-0.1)
H3	0.0078	0.0078 (0.0039-0.0078)	0.1
H27	0.0078 (0.0078-0.0156)	0.0078 (0.0078-0.0156)	0.1
H44	0.0078	0.0078 (0.0039-0.0078)	0.1
H65	0.0078 (0.0078-0.0156)	0.0078 (0.0078-0.0156)	0.1
D39N	0.0078	0.0078 (0.0039-0.0078)	0.1
NCTC7466	0.0078 (0.0078-0.0156)	0.0078 (0.0039-0.0078)	0.1

Supplementary Table 2. Transformation efficiency of six D39 strains. Data are presented as mean transformation efficiency from three independent experiments, each containing two technical replicates per strains. P values are from one-way ANOVA with Dunnett's multiple comparisons test, with NCTC7466 as the control strain.

Strain	Transformation efficiency	Standard deviation	P-value vs NCTC7466
H1	2.37×10^{-3}	0.0002484	0.1252
H3	5.76×10^{-3}	0.0005558	0.2622
H27	2.16×10^{-3}	0.0006583	0.2937
H44	2.72×10^{-3}	0.0000584	*0.0426
H65	4.42×10^{-4}	0.0003575	0.0842
D39N	2.84×10^{-3}	0.0004903	0.3523
NCTC7466	4.00×10^{-3}	0.0000096	
